# Clinical and Pathological Findings Associated with Mycobacteriosis in Captive Syngnathids

**DOI:** 10.3390/ani12233259

**Published:** 2022-11-23

**Authors:** Estefanía Montero, Carlos Rojo-Solís, Noelia de Castro, Miguel Fernández, Valentín Pérez, Juan M. Corpa, Joaquín Ortega

**Affiliations:** 1 Pathology Group, PASAPTA, Facultad de Veterinaria, Universidad Cardenal Herrera-CEU, CEU Universities, C/Tirant lo Blanc 7, Alfara del Patriarca, 46115 Valencia, Spain; 2 Veterinary and Laboratory Service, Oceanogràfic, Ciudad de las Artes y las Ciencias, C/Eduardo Primo Yúfera (Cientific) 1B, 46013 Valencia, Spain; 3 Veterinary Services, Aquarium Finisterrae, Paseo Marítimo Alcalde Francisco Vázquez 34, 15002 La Coruña, Spain; 4 Departamento de Sanidad Animal, Instituto de Ganadería de Montaña (CSIC-ULE), Facultad de Veterinaria, Universidad de León, 24071 León, Spain

**Keywords:** granuloma, mycobacteriosis, *Mycobacterium chelonae*, *Mycobacterium fortuitum*, *Mycobacterium marinum*, pathology, pipefish, sea dragon, seahorse, syngnathids

## Abstract

**Simple Summary:**

Syngnathids are teleost fish that include seahorses (*Hippocampus* ssp.), pipefishes (*Syngnathus* spp.), common seadragons (*Phyllopteryx taeniolatus*) and leafy seadragons (*Phycodurus eques*). Some species are currently threatened. Reproduction and maintenance in aquariaare complicated and highly contagious diseases, such as mycobacteriosis, can trigger numerous casualties. The most frequently common species observed in fish are *Mycobacterium marinum*, *M. fortuitum* and *M. chelonae*, which belong to the group of non-tuberculous mycobacteria. Stress factors, poor water quality, bad management and high population densities are associated with a higher prevalence of this disease. This study describes the clinical signs and granulomatous lesions associated with mycobacteriosis in syngnathids. *M. fortuitum* and *Mycobacterium* spp. were isolated in 4 and 14 syngnathids, respectively. Seven samples were positive against *M. chelonae* and *M*. *marinum* common primers. Considering the scarcity of pathological studies in syngnathids, these findings can help to improve the clinical management and survival of these animals in captivity.

**Abstract:**

Mycobacteriosis is an important disease that affects captive and wild aquatic fish. Syngnathids are susceptible to infection by non-tuberculous mycobacteria. The aim of this study was to describe clinical signs, and macroscopic and histological lesions in 25 syngnathids and the molecular characterization of the causative mycobacteria. Clinical presentation ranged from sudden death to non-specific signs, including anorexia, poor body condition, weight loss and marked dyspnea with increased respiratory effort and rate. Gross lesions were mostly ulcers on the tail and small white nodules in the liver, coelomic cavity and inside the eye. The most affected organs were gills, liver, intestine and coelomic mesentery. Microscopic lesions consisted of areas of multifocal to diffuse granulomatous inflammation and bacterial emboli with numerous intralesional acid-fast bacilli. Epithelioid cells, multinucleated giant cells, lymphocytes and fibrous connective tissue, which are commonly observed in granulomatous inflammation, were not observed here. In the real-time PCR, *M. fortuitum*, *M. chelonae* and *M. marinum* common primers, *Mycobacterium* spp. were detected in 4, 7 and 14 individuals, respectively. In addition, this is the first description of mycobacteriosis found in *Syngnathus acus*.

## 1. Introduction

Mycobacteriosis is a potentially fatal bacterial disease in wild and captive fish caused by the bacteria of the non-tuberculous mycobacteria (NTM) group [1]. Mycobacterium spp. belong to the family Mycobacteriaceae, order Actinomycetales [2]. They are non-motile, aerobic and pleomorphic bacilli usually identified with the Ziehl–Neelsen stain [3]. Many fish species are susceptible to severe fatal NTM infections [2,4]. Currently, *Mycobacterium marinum*, *M fortuitum*, *M chelonae* are the most common species in captive aquatic animals [2], together with *M triviale*, *M avium*, *M abscessus* and *M peregrinum*, which have been regularly reported in ornamental fish [5,6,7,8]. Generally, in fish species, the histological presentations are granulomas, formed by an occasional necrotic core bordered by a zone of epithelioid macrophages, few giant multinucleated cells, and lymphocytes. All these are surrounded by thin bands of fibrous connective tissue [9,10]. Mycobacteriosis is reported to be an acute to chronic disease in captive syngnathids, such as seahorses, sea dragons and pipefish, and is one of the main causes of mortality in these species [11]. In addition, syngnathids present an atypical granulomatous lesion compared to other teleosts with necrosis and large numbers of macrophages, but without giant cells, lymphocytes, epithelioid cells or fibrosis [3,12]. However, very few reports confirm these findings. Nowadays, all seahorse species are included in Appendix II of endangered species by CITES, which restricts the legal import and export of these animals [13,14]. Wild syngnathid populations currently face many threats, including habitat loss, pollution, climate change, competition with invasive species and direct exploitation in the form of overfishing and by-catches [15,16]. Given these circumstances, profound concern is voiced about their populations’ long-term viability in the wild, with some species classified as critically endangered, endangered, vulnerable and near-threatened by the IUCN [15,16,17]. Captive breeding and re-introduction programs have been applied for the last few decades in an attempt to reverse this trend [18]. However, the captive breeding of many species of syngnathids remains a challenge due to their susceptibility to diseases and management issues such as stress control, water quality management and high population densities [19,20]. As very little is known about the development of diseases in these animals in captivity, the objectives of this work were to: (i) provide a detailed description of the clinical and pathological findings associated with mycobacterial infections; (ii) identify the causative etiological agents.

## 2. Materials and Methods

### 2.1. Case Selection 

A search was conducted in the Veterinary Pathology Service facilities of the CEU Cardenal Herrera University (Valencia, Spain) from 2010 to 2022. All the animals were necropsied, and a full histological examination was performed. Out of 393 sygnathids, 25 showed histological lesions and special stains results consistent with mycobacteriosis and were included in this study. The selected syngnathids were referred from the Oceanogràfic Aquarium (Valencia, Spain) and the Finisterrae Aquarium (La Coruña, Spain).

### 2.2. Animal Husbandry Conditions

Animals were maintained in aquarium display tanks or quarantine tanks in the differentaquaria, provided with artificial decoration and substrates. Water quality was warranted by mechanical and biological filtration systems, and was monitored daily by a multiparametric physical analysis, including temperature, pH, salinity, density, dissolved oxygen (DO) and oxidation reduction potential (ORP); biochemical analyses twice weekly, including ammonia (NH3), nitrite (NO2) and nitrate (NO3) concentrations. Water salinity and temperature settings varied depending on species, based on the normal characteristics found at their original geographical distribution; lighting was provided with LED lamps in a 12-h light-darkness cycle. Water disinfection was achieved with UV or ozone. Total aerobic mesophilic bacteria and *Vibrio* sp. concentrations were determined once every 3 months using standardized methods for water microbial analyses. 

The food offered also varied with species but was composed mainly of live or frozen mysids (*Mysis* sp.) and live or frozen artemia (*Artemia salina*). Live mysids were collected from naturally occurring salt evaporation ponds or other tanks in aquaria, while artemia was reared from commercially available eggs hatched in the facilities and used in the larval (nauplii) or adult stages depending on syngnathid age and size. Artemia was enriched with fatty acids or garlic extract, offered alternately toward a more complete diet. Healthy and sick animals were checked daily by the aquarium staff.

### 2.3. Necropsy and Histology

All animals used on this study were deceased syngnathids, on which a complete external examination, including skin scrapes and gills biopsy, was carried out by the referring veterinarians at the Oceanogràfic Aquarium, Valencia, Spain (C. Rojo-Solís) and the Finisterrae Aquarium, A Coruña, Spain (N. Castro).

Animals were classified as adults or juveniles based on the snout to tail-tip length and sex determined based on the presence/absence of brood pouch. In this study, all animals were adults (over 3 cm of snout to tail-tip length), the coelomic cavity was opened and the whole carcass was immersed in 10% neutral-buffered formalin for 24 h. Time from death to post-mortem examination and fixation was less than 12 h. All the syngnathids fixed specimens were sent to the Veterinary Pathology Service at the CEU Cardenal Herrera University (Valencia, Spain). After fixation, specimen necropsy was performed. The skeleton was decalcified using 4% nitric acid for 1–4 h depending on the animal’s size. Animals measuring less than 5 cm in length decalcified within 1 h. In contrast, animals that exceeded 5 cm in length up to 15 cm were decalcified for 2–4 h. All the tissues were processed routinely, embedded in paraffin and hematoxylin and eosin (H & E)-stained. Additional stains on selected tissues, including acid-fast Ziehl–Neelsen (ZN) and Gram stains, were performed. The histological sections were examined by light microscopy. Animals were considered infected if acid-fast bacilli were found during the microscopic examination. The most common diagnostic tool for mycobacterioses in fish involves culture. However, the culture in this study is not possible because the samples were fixed in formalin.

### 2.4. Molecular Identification

DNA was extracted from ZN-positive and paraffin-embedded samples with Maxwell^®^ RSC FFPE Plus DNA Kit (Promega^®^, Madison, WI, USA) following manufacturer’s instructions for later testing with mycobacteria-specific Real Time (RT)-PCR assays. Extracted DNA was diluted at 50 ng µL^−1^. Products were stored at −20 °C prior to mycobacteria detection. The employed primers were designed for the screening of *Mycobacterium avium* subsp. *paratuberculosis* IS900; *Mycobacterium avium* subsp. *avium* IS901; *Mycobacterium marinum*, *M chelonae* and *M fortuitum* shared 16S-23S internal transcribed spacer (ITS); *M lepraemurium* and *M fortuitum* ITS sequences. PCR reactions were performed using 20 μL of the SYBR^®^ Green PCR Master Mix (Applied Biosystems, Foster City, CA, USA), 0.2 µM of each primer, and 50 ng of diluted DNA samples in the ABI 7500 fast Real-time PCR system (Applied Biosystems^®^, Waltham, MA, USA) with the following parameters 95 °C for 30 s (sec) (×1); 95 °C for 5 sec (×1); 60 °C for 34 sec (×40); 95 °C for 15 sec and 60 °C for 1 min and cold for store (Table 1).

The RT-PCR results were analyzed using 7500 Software v2.0.6 (Applied Biosystems^®^, Waltham, MA, USA). Furthermore, positive results were considered when dissociation peak (Tm) was 89.1 ± 1.5 °C and threshold cycles (Ct) were < 37. Positive samples from ruminants, bird, Nile crocodile, environment and cat have been included for each etiology sought.

## 3. Results

### 3.1. Clinical Signs and Management

The clinical presentation in the affected animals varied from sudden death without previous symptoms (n = 9 animals) to nonspecific clinical signs (n = 16 animals), including anorexia, poor body condition and weight loss, marked dyspnea with increased respiratory effort and rate and, finally, prostration and death. As making an ante-mortem diagnosis is difficult in these species, treatment was attempted in some individuals under the critical condition with broad-spectrum antibiotics (ceftazidime), vitamin supplements (vitamin B complex) and corticoids (dexamethasone), but was unsuccessful. In the moderately dyspneic individuals, water hyperoxygenation was performed. Nonetheless, animals died a few weeks later. Animals from both sexes were evenly affected.

### 3.2. Gross and Histopathological Findings 

**Figure 1:**
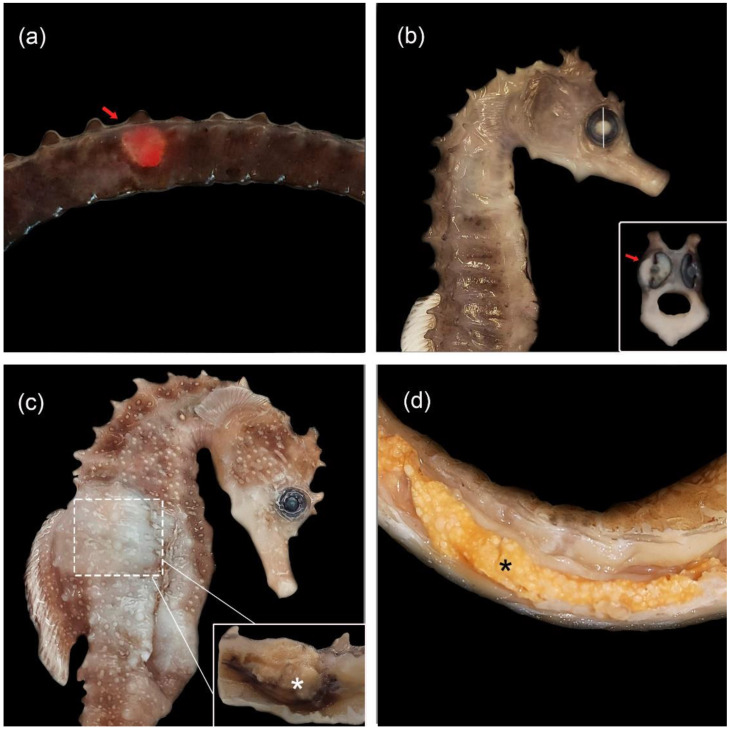
Gross lesions of syngnathids with mycobacteriosis. (**a**) *Hippocampus reidi* with a 4 mm diameter red ulcer on the tail (arrow). (**b**) Seahorse *H. guttulatus* with mild exophthalmia and opaque appearance of the right eye. Inset: Cross-section of the white eye shows a 3 mm retrobulbar white nodule displacing the eye (arrow). (**c**) *H.s guttulatus* with white and raised skin due to the presence of a 1 cm nodule in the coelomic cavity (arrow). Inset: Cross-section reveals a 1 cm nodule with raised contour and firm consistency originating from coelomic cavity the wall (asterisk). (**d**) Weedy seadragon (*Phyllopteryx taeniolatus*) with 1 mm white multifocal to coalescing nodules in the liver (asterisk).

Upon external examination of unfixed specimens and necropsy, only eight (32%) of the 25 animals presented macroscopic findings. Gross findings consisted of large amounts of mucus in gills (n = 8), poor body condition due to loss of fatty deposits (n = 7), soft exoskeleton (n = 4) and 2–5 mm ulcers on tails (n = 4) (Figure 1a). Variable sized nodules were observed in the eye (n = 2), coelomic cavity (n = 1) and the liver (n = 1). In the eye, white nodules (1–2 mm) were observed inside the eye to cause exophthalmia (Figure 1b). One seahorse showed a whitish, irregular and slightly raised area of skin at the level of the coelomic cavity (Figure 1c). A longitudinal section revealed a 1 cm diameter nodule attached to the coelomic wall that raised the skin (Figure 1c; insert). In the liver, 1–2 mm nodules with a multifocal to coalescent distribution and raised contour were also observed, which deepened in the section (Figure 1d). In all the other infected animals (n = 17), no gross lesions were observed. 

**Figure 2:**
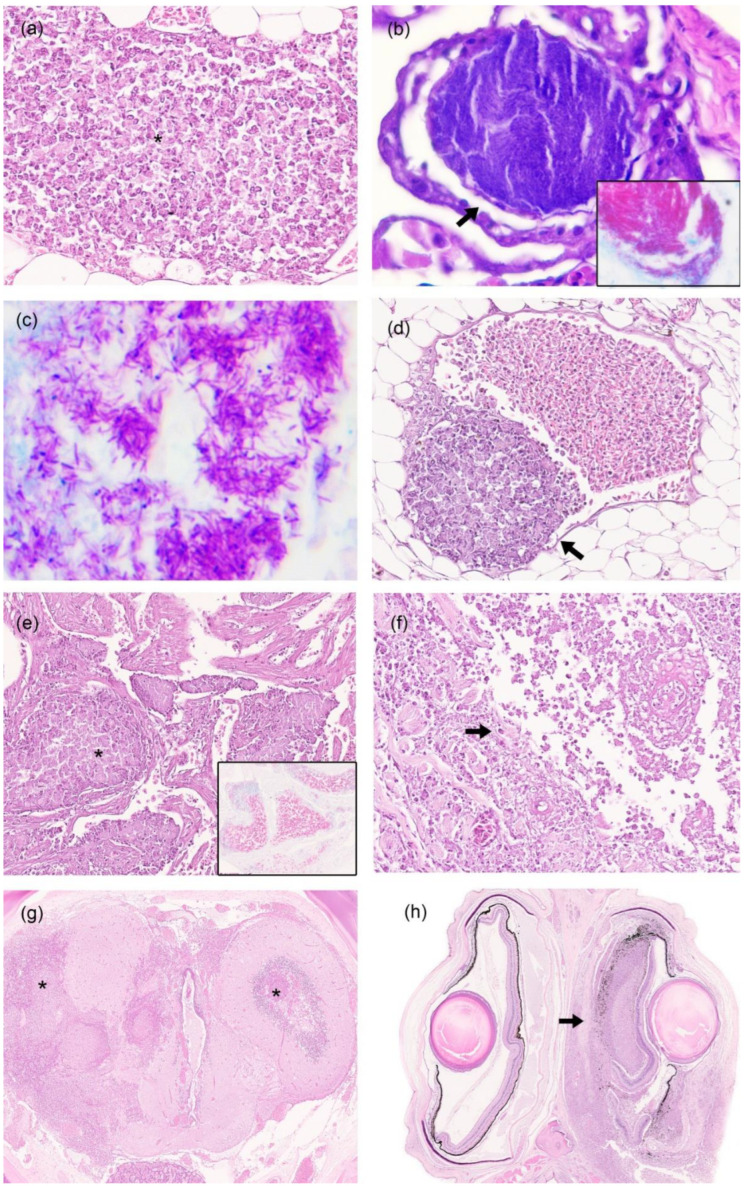
Microscopic lesions of syngnathids with mycobacteriosis. (**a**) Coelomic mesentery. *Phyllopteryx taeniolatus* with nodular to diffuse granulomatous inflammation and necrotic debris (asterisk). H & E stain 40×. (**b**) Gills. *Syngnathus acus* with lamellar capillary distended and occluded by bacterial emboli (arrow). H & E; 60×. (**c**) Liver with numerous acid-fast bacteria-laden macrophages. Ziehl–Neelsen stain; 60×. (**d**) Mesenteric tissue. *Syngnathus acus* with a thrombus formed by macrophages adhered to the wall of a mesenteric tissue vessel (arrow). (**e**) Heart. *Syngnathus acus* with bacterial emboli in the lumen of the heart ventricle (asterisk). H & E; 10×. (**f**) *Hippocampus reidi* with replacement of collagen fibers in the skin by macrophages and necrosis (arrow). H & E stain; 20×. (**g**) Brain. *Hippocampus guttulatus* showing an asymmetric inflammation in cerebral hemispheres composed of macrophages and necrotic debris that replace the neuropil (asterisk). H & E stain; 2×. (**h**) Eye. *Hippocampus guttulatus* with asymmetrical eyes due to the presence of periocular granulomatous inflammation that infiltrated and ruptured the right eye (arrow). H & E stain; 2×.

All the animals in the study (n = 25) showed histological lesions located in gills (n = 22), the liver (n = 18), intestine (n = 15), coelomic mesentery (n = 15), kidney (n = 9), heart (n = 8), skeletal muscle (n = 4), skin (n = 4), brain (n = 3), eye (n = 2), gas gland (n = 1), swim bladder (n = 1) and ovary (n = 1) (Table 2). In the aforementioned organs, histological lesions were similar and characterized by a nodular to diffuse granulomatous inflammation composed of acid-fast bacteria-laden macrophages admixed with cellular debris and bacterial emboli (Figure 2a–c). Nodules were expansive and poorly demarcated due to the absence of external fibrous tissue. No multinucleated giant cells, lymphocytes or epithelioid cells were observed. Gills were the most affected organ, where granulomatous inflammation with a nodular pattern was observed, and lamellar capillaries were distended and occluded by bacterial nodules (Figure 2b). In addition to emboli, thrombi were observed to produce vasculitis in the coelomic mesentery (Figure 2d). The hearts of all the animals presented numerous bacterial emboli, which were even observed both with routine stains (H & E) and with ZN within the heart chambers (Figure 2e). Skin presented granulomatous inflammation with a more diffuse pattern (Figure 2f). In the three cases in which the brain was affected, the presence of asymmetric inflammation in both cerebral hemispheres replaced the nervous tissue (Figure 2g). In two eyes, asymmetry was observed due to the presence of inflammation, which affected the periocular area that compressed and infiltrated eyes. Inflammation extended to the choroid and cornea and caused the retina to rupture (Figure 2h).

### 3.3. Bacterial Identification by PCR

Of the total of 25 samples studied by RT-PCR, seven samples were positive after using *M. chelonae*, *M. fortuitum* and *M. marinum* common 16-23S primers. Among them, four were also confirmed positive after the amplification of *M. fortuitum* primers. There were also unspecific samples (n = 14), only designated as *Mycobacterium* sp. since late Ct value was seen both for *M. avium avium* and *M. marinum* primers. None of the studied etiologies were found in the rest of the animals (n = 10) (Table 2). Positive results were considered when dissociation peak (Tm) was 89.1 ± 1.5 °C and threshold cycles (Ct) were < 37 for each target and amplification plot.

## 4. Discussion

The infections caused by non-tuberculous mycobacteria are common throughout aquatic species, probably due to their ubiquitous presence, particularly in the aquatic environment [2]. Although all fish species can be susceptible to mycobacteriosis, members of the families Anabantidae, Characidae, Cyprinidae, Cichlidae and Syngnathidae are most commonly reported [3,18,21]. 

Syngnathids are highly valuable teleosts and mycobacteriosis is an important disease [18]. In our study, the presence of animals with mycobacteriosis accounted for only 6.4% of the cases (25 out of 393), which is a much lower rate than similar previously reported studies (15%, 25 out of 172) [11]. This difference could be related to the tank conditions in the different aquaria because water treatment with UV or ozone can decrease the prevalence of *Mycobacteria*.

The species of syngnathids in which mycobacteriosis was observed included *H. guttulatus*, *H. reidi*, *H. abdominalis*, *S. acus*, *S. biaculeatus* and *P. taeniolatus*. To the authors’ knowledge, mycobacteriosis has not been previously described in *S. acus*, which would mean that this is its first description in this species.

The clinical presentation observed in this study was variable and appeared in two main manifestations: an acute presentation without apparent clinical signs causing sudden death and a chronic course. The chronic course was associated with weight loss, anorexia, marked dyspnea with increased respiratory effort and rate, and prostration. Variability of clinical signs between species has been shown and may be related to the immunity, bacterial quantity, location and severity of the lesion. As these clinical findings are very nonspecific, the diagnosis of mycobacteriosis cannot be made, or even suspected, without performing a necropsy and histological studies. Some of the above-cited clinical signs in syngnathids, including lethargy, poor appetite, abdominal swelling, ascites, scale loss and dermal ulcerative necrosis, exophthalmia, blindness and pale gills, as well as skeletal deformities, such as spinal curvature or stunted growth, are similar in other teleosts with mycobacteriosis [2]. It is important to highlight the importance of the acute clinical presentation observed in our study, which is contrary to what occurs in other animal species. Syngnathids that did not show clinical signs are likely to be associated with an acute presentation, whereas animals that did show clinical signs are likely to be associated with a chronic presentation. Animals with an acute presentation presented moderate to severe bacterial load affecting few organs. On the other hand, animals with chronic presentation showed a moderate bacterial load affecting many organs. Therefore, there could be an association between the clinical signs, the course of the pathology (acute or chronic) and the bacterial load. In a previous study, the authors hypothesized that mycobacteriosis in syngnathids involving large numbers of bacilli are more consistent with acute, fulminant and septicemic infection, as opposed to the relatively small numbers of bacteria and the chronic granuloma formation commonly found in other teleosts [3]. 

Macroscopic findings were also variable in this study and only 32% of the animals (8 out of 25) presented some type of gross lesion. The most frequent findings were excessive mucus in gills, followed by poor body condition and a soft exoskeleton, lesions that have not been described in previous articles of syngnathid mycobacterioses. However, ulcerative skin lesions, one of the gross lesions more commonly associated with mycobacteriosies [11], was found in only 16% (4 out of 25) of our fish. The higher number of lesions in gills than skin could justify a predominant septicemic pattern against a more local dermal pattern. This systemic dissemination would reinforce the acute course of the disease in some animals.

The histological lesions were located more frequently in gills, the liver, intestine and coelomic cavity, and were characterized by the formation of variable sized areas of macrophage accumulation with cellular debris. Multinucleated giant cells, epithelioid macrophages, lymphocytes and surrounding connective tissue were not observed as in other teleosts [27]. For this reason, this microscopic finding has been previously named atypical granulomas [3,20]. Currently, it is unclear as to whether these atypical lesions are the result of an ineffective cell-mediated immune response, the virulence of the pathogen or a combination of both. In a previous report, interferon-gamma (IFN-y), the key element of the adaptive cellular immune response against mycobacteria, was not identified and could be the reason for the susceptibility and atypical pathological lesions observed in syngnathids [3]. These observations suggest that a genetic basis may be responsible for the nature of the infections and lack of granuloma formation in syngnathids [3]. In our opinion, these mycobacteria induce a nodular to diffuse granulomatous inflammatory response instead of well-organized classic multifocal granulomas with a necrosis core surrounded by lymphocytes, macrophages, giant cells and an external fibrous capsule. 

In a study of mycobacterioses in a population of Atlantic jack mackerel (*Trachurus trachurus*), three types of granulomas were described: cellular granuloma, necrotic-core granuloma and lamellar granuloma [11]. The cellular granuloma, composed mainly of macrophages and hypothesized as an immature phase, are a more similar finding to those observed in this study. 

In this work, mycobacteria were present in two forms: inside macrophages or forming emboli in the heart and the vessels of other organs. It is important to highlight from our work the presence of free mycobacteria without having to travel through macrophages as in most species. This finding is indicative of septicemia, which produces an acute course of the disease with hardly any macroscopic lesions.

Differentiating NTM species by gene sequencing can be challenging. In fact, the 16s rRNA, hsp65 and rpoB gene sequences have a similarity of 97% [28]. However, other studies [29] found that the 16s rRNA is useful for identifying NTM species and differentiating them from the rpoB gene of *Mycobacterium* complex by PCR. Previous literature describes those seahorses, seadragons and pipefish commonly suffer significant losses primarily due to *M chelonae* [3,11,12]; however, that was not found in this study, leading to the conclusion that it seems not be an important pathogen in the aquatic facilities where the studied animals were maintained. In other captive and free aquatic animals, the isolation of *M marinum* is more common [2], in accordance with this study, where DNA from this mycobacterium was identified. An outstanding finding has been the identification of *M fortuitum* by RT-PCR in four animals. While this mycobacterial species has not been previously identified in seahorses, it is a common pathogen for different species of teleosts [6,27,30]. Mycobacteriosis was confirmed in the rest of the individuals due to the presence of ZN acid-fast bacilli, but the species were not identified. Only a mycobacterium was identified, belonging to the *Mycobacterium* genus with late cycle threshold value (equal or above 37) for Maa, Map and *M marinum*-*M fortuitum*-*M chelonae* common primers. The possibility that *M fortuitum* was infecting these animals cannot be discarded, since this bacterium has been considered to be NTM [31]. The fact that in some cases the melting curve was late would suggest that the number of mycobacteria in the particular section examined was low, or that tissue handling, particularly DNA fragmentation as a result of the use of formalin and the time of fixation, could have affected the PCR results [32,33]. This could be caused because the tissues were fixed in formalin for a long period and, consequently, the DNA could be degraded. On the other hand, the mycobacteria found may have been transmitted by water [34] and, according to the results of our study, the possibility that the mycobacteria were present in the water tanks of the Syngnathids should be highlighted. Further studies with the aim of investigating the possible origin of the mycobacterial contamination should be conducted. 

## 5. Conclusions

The main finding in our study was the presence of granulomatous inflammation affecting several organs in all species of syngnathids and numerous bacteria emboli. It is important to highlight the acute and septicemic course of mycobacteria in *Syngnathidae* and the absence of external lesions in many animals. Moreover, the clinical signs observed in some animals were very nonspecific as often described in cases of mycobacteriosis affecting aquarium fish. All these factors make the clinical management of this disease very complicated and might be the reason why it is so difficult to eliminate the mycobacteriosis in captive fish. Thus, a necropsy and posterior histological analysis are fundamental to diagnose the disease.

Unlike other studies, *M fortuitum* was the most common mycobacteria isolated in syngnathids. As no cases of mycobacteriosis have been reported in *Syngnathus acus*, this would be the first description in this species.

Due to the scarcity of pathological studies in these species, these findings can contribute to the prevention, diagnosis, and treatment of diseases, and favor the conservation and welfare conditions of syngnathids in captivity.

## Figures and Tables

**Table 1:** Primers designed for the different sequences used in this study. Reverse and primer concentrations: 0.2 µM. PCR master mix final volume: 20 μL. Amplification product size: 254-bp.

Species (Gene)	Primer Design
*Mycobacterium avium* subsp. *paratuberculosis* (IS900) ^[21]^	F:GATCGGAACGTCGGCTGGTCAGG
	R:GATCGCCTTGCTCATCGCTGCCG
*Mycobacterium avium* subsp. *avium* (IS901) ^[22]^	F:AAGCCGAGGTGGTGTATGT
	R:AGCGAAGATGGCGGTGAGCAT
*Mycobacterium marinum* (16S-23S ITS) ^[23]^	F:CACCACGAGAAACACTCCAA
	R:ACATCCCGAAACCAACAGAG
*M marinum*-*M fortuitum*-*M chelonae* (16S-23S ITS) ^[24]^	F:GCTGGATCACCTCCTTTCTA
	R:AGATGCTCGCAACCACTAT
*M lepraemurium* (16S rRNA) ^[25]^	F:GAATATTGCACAATGGGCGCAG R: AAACCCGGACCTTCGTCGATA
*oneM fortuitum* (16S-23S ITS) ^[26]^	F:GACTGCCAGACACACTATTGG
	R:GTGAGACCACACGATTCTGC

Superscripts [21,22,23,24,25,26]: references of each specie’s primer design.

**Table 2:** Animals, samples studied and mycobacterial identification by PCR.

Syngnathids		Species	Sex	Date of Sampling	Acute or Chronic Presentation	Clinical Signs and Gross Changes	Affected Tissue	Bacterial Load	Identified Microorganism
1	Seahorse	*Hippocampus guttulatus*	Female	03/11/2020	Chronic	Dyspnea mucus in gills, tail ulcer	Liver, skin, kidney, gills, intestine, heart	Mild to moderate	*M chelonae*, *M marinum*
2		*Hippocampus guttulatus*	Female	30/09/2020	Acute	Sudden death	Liver, intestine, heart	Moderate	*M fortuitum*
3		*Hippocampus guttulatus*	Male	19/02/2021	Chronic	Dyspnea, mucus in gills	Liver, heart, intestine, gills	Moderate	*M chenolae*, *M marinum*
4		*Hippocampus guttulatus*	Male	17/02/2021	Chronic	Dyspnea, mucus in gills tail ulcer, poor body condition	Liver, intestine, skeletal muscle, kidney, skin, gills, heart, blood vessel	Moderate	*M chelonae*, *M marinum*
5		*Hippocampus guttulatus*	Female	17/03/2021	Chronic	Dyspnea, mucus in gills, white eye, exophthalmia	Liver, intestine, kidney, gills, eye	Mild to moderate	*M fortuitum*
6		*Hippocampus guttulatus*	Male	24/05/2021	Acute	Sudden death	Liver, intestine, gills, heart	Moderate	*M chenolae*, *M marinum*
7		*Hippocampus guttulatus*	Male	05/09/2021	Chronic	Tail ulcer, soft exoskeleton, mucus in gills	Liver, intestine, gills, heart, skin	Mild to moderate	*Mycobacterium* sp.
8		*Hippocampus guttulatus*	Male	11/08/2021	Chronic	Coelomic cavity mass, poor body condition	Liver, intestine, skeletal muscle, kidney, gills	Mild to moderate	*M chenolae*, *M marinum*
9		*Hippocampus guttulatus*	Male	25/08/2021	Chronic	White eye, exophthalmia, mucus in gills	Liver, intestine, skeletal muscle, kidney, skin, heart, gills, blood vessel, brain	Moderate	*M chenolae*, *M marinum*
10		*Hippocampus reidi*	Female	08/12/2020	Acute	Sudden death	Gills, blood vessel	Moderate to severe	*Mycobacterium* sp.
11		*Hippocampus reidi*	Female	13/01/2021	Acute	Sudden death	Liver, gills	Moderate	*Mycobacterium* sp.
12		*Hippocampus reidi*	Male	02/02/2021	Chronic	Poor body condition	Liver, intestine, gills, blood vessel	Moderate	*Mycobacterium* sp.
13		*Hippocampus reidi*	Male	14/4/2021	Chronic	Soft exoskeleton	Liver, intestine, skeletal muscle, gills, brain	Moderate	*Mycobacterium* sp.
14		*Hippocampus reidi*	Female	12/03/2021	Chronic	Poor body condition	Liver, intestine, kidney, gills, brain	Mild to moderate	*Mycobacterium* sp.
15		*Hippocampus reidi*	Female	18/08/2021	Chronic	Tail ulcer, soft exoskeleton, mucus in gills	Kidney, skin, gills, brain, blood vessel	Moderate	*Mycobacterium* sp.
16		*Hippocampus reidi*	Female	16/09/2021	Chronic	Dyspnea, mucus in gills	Liver, kidney, gills, brain	Moderate	*Mycobacterium* sp.
17		*Hippocampus abdominalis*	Male	18/05/2018	Acute	Sudden death	Liver, intestine, gills	Moderate	*Mycobacterium* sp.
18		*Hippocampus abdominalis*	Female	11/07/2018	Chronic	Poor body condition	Gills, eye, swim bladder	Mild to moderate	*Mycobacterium* sp.
19		*Hippocampus abdominalis*	Female	26/02/2021	Acute	Sudden death	Gills, blood vessel	Moderate to severe	*Mycobacterium* sp.
20		*Hippocampus abdominalis*	Male	23/04/2021	Acute	Sudden death	Gills, blood vessel	Moderate	*M fortuitum*
21	Pipe fish	*Syngnathus acus*	Female	11/07/2018	Acute	Sudden death	Gills, blood vessel	Moderate	*Mycobacterium* sp.
22		*Syngnathus acus*	Female	05/04/2021	Chronic	Soft exoskeleton	Liver, intestine, gills, gas gland	Mild to moderate	*Mycobacterium* sp.
23		*Syngnathoides biaculeatus*	Male	16/07/2018	Acute	Sudden death	Gills, blood vessel	Moderate	*M chenolae*, *M marinum*
24	Seadragon	*Phyllopteryx taeniolatus*	Female	8/07/2018	Chronic	Poor body condition	Liver, kidney, ovary	Mild to moderate	*Mycobacterium* sp.
25		*Phyllopteryx taeniolatus*	Female	25/01/2021	Chronic	Poor body condition	Liver, intestine, gills, heart, kidney	Mild to moderate	*M fortuitum*

## Data Availability

The data reported in this paper were generated specifically for the study and they are showed in Table 2.
